# The Racial Disparities in the Epidemic of Metabolic Syndrome With Increased Age: A Study From 28,049 Chinese and American Adults

**DOI:** 10.3389/fpubh.2021.797183

**Published:** 2022-02-01

**Authors:** Ruiying Zhang, Jie Sun, Chaofan Wang, Xiangtuo Wang, Pei Zhao, Yucong Yuan, Hu Ai, Qi Zhou

**Affiliations:** ^1^Department of Nephrology, Harrison International Peace Hospital, The People's Hospital of Hengshui City, Hengshui, China; ^2^Graduate School of Peking Union Medical College, Chinese Academy of Medical Sciences, Beijing, China; ^3^School of Public Health, Capital Medical University, Beijing, China; ^4^Department of Cardiology, National Center of Gerontology, Institute of Geriatric Medicine, Beijing Hospital, Chinese Academy of Medical Sciences, Beijing, China; ^5^The Key Laboratory of Geriatrics, National Center of Gerontology of National Health Commission, Beijing Institute of Geriatrics, Institute of Geriatrics, Beijing Hospital, Chinese Academy of Medical Sciences, Beijing, China

**Keywords:** metabolic syndrome, age, ethnic disparity, primary prevention, public health

## Abstract

**Background:**

Previous studies have revealed ethnic disparities in the prevalence of metabolic syndrome (MetS); however, the literature regarding aging-related patterns of disparities in MetS and its components remains limited.

**Methods:**

This cross-sectional study recruited 28,049 subjects, consisting of one Chinese race and three American races, 18–85 years of age, from the National Health and Nutrition Examination Survey (NHANES, 1999–2018) of the United States, and the Guangdong Gut Microbiome Project (GGMP, 2018) of China. MetS was defined in accordance with the National Cholesterol Education Program Adult Treatment Panel III. A modified sliding-window-based algorithm was used to depict the trajectories of the prevalence of MetS with increased age. Logistic regression analysis was performed to investigate associations between MetS and its components.

**Results:**

The prevalence of MetS increased non-linearly with age, with growth speed reaching its maximum at approximately 40–50 years. Chinese subjects exhibited a lower prevalence of MetS than non-Hispanic whites, non-Hispanic blacks, and Mexican Americans in all age groups. The two most prevalent components in Chinese subjects were reduced high-density lipoprotein cholesterol levels (42.0%) and elevated blood pressure (49.5%), and elevated triglyceride levels (36.3–49.5%) and abdominal obesity (55.8–55.9%) in Americans. Before 40 years of age, the top two MetS-associated components were abdominal obesity and elevated triglyceride levels in all races, while after 40 years, the prominent associations between MetS and its components varied among the different races and age groups.

**Conclusions:**

Although racial disparities in the epidemic of MetS varied with increased age, abdominal obesity and elevated triglyceride levels were the top two MetS-associated components in all younger adults of different races.

## Introduction

Metabolic syndrome (MetS), characterized by the cluster of obesity, elevated blood pressure (BP), dyslipidemia, and hyperglycemia, is a strong risk factor for cardiovascular disease and type 2 diabetes mellitus. The prevalence of MetS had reached approximately one-quarter of the global population according to an estimate in 2018; as such, the prevention of MetS has become a global initiative (1).

Insulin resistance and abdominal obesity have generally been regarded to be the key components of MetS that require primary control (1). However, previous studies have reported inconsistent results across ethnicities. The most two prevalent components were reduced high-density lipoprotein cholesterol (HDL-c) levels and hypertension in Brazilian and Indonesian populations (2, 3), abdominal obesity and hypertension in European countries (4), and hypertension and dyslipidemia among the Chinese (5), whereas abdominal obesity and hypertension contributed most to MetS in Malaysian populations (6). It remains challenging for experts to determine the underlying causes of MetS in populations comprising different ethnicities, as well as which risk factors should be controlled first in primary preventive measures against the development of MetS.

The prevalence of MetS increases with age and the prominent components are also different between young adults and the elderly. The increase in MetS prevalence from those 19–39 years to 60–78 years of age was reported to be almost five-fold in European populations (7). Elderly individuals in the United States also demonstrated a prevalence of MetS ~2 times higher than subjects <40 years of age (8). Moreover, aging has been found to be a strong risk factor for blood glucose impairment independent of obesity and exhibited a significant influence on other MetS components (9, 10). Thus, we hypothesized that the aforementioned disparity between ethnicities/races, regions, or studies may be significantly affected by non-stratified age groups. The prominent components of MetS may also be influenced by their different growth rate with age.

Although the characteristics of MetS vary among populations of different ethnicities, only a limited number of studies have documented disparities across stratified age categories. The present study aimed to evaluate how the prevalence of MetS changed with age among non-Hispanic white, non-Hispanic black, Mexican American, and Han Chinese subjects, and to investigate whether the associations between MetS and its components change with aging across the four races.

## Materials and Methods

### Data Sources

Data for this survey were collected from two datasets: the National Health and Nutrition Examination Survey (NHANES, 1999–2018) of the United States; and the Guangdong Gut Microbiome Project (GGMP, 2018) of China (11). Data from both the NHANES and the GGMP are sampled by randomized strategies and consist of demographic, health-related questionnaires, as well as physical examinations. The two survey protocols were approved by the Institutional Review Board of the Centers for Disease Control and Prevention (Atlanta, GA, USA) and the Ethical Review Committee of the Chinese Centre for Disease Control and Prevention.

The most three prominent races (non-Hispanic white, non-Hispanic black, and Mexican American) in NHANES and one race (Han Chinese) in the GGMP were included in this study. All of the enrolled samples were required to have age, sex, and components of MetS, which consist of waist circumference (WC), fasting plasma glucose (FPG), systolic BP (SBP), diastolic BP (DBP), triglycerides (TG), and HDL-c. Subjects reported to have cancer or malignant tumors were excluded. Ultimately, 10,327 non-Hispanic white, 5,674 non-Hispanic black, 5,134 Mexican American, and 6,914 Han Chinese subjects were included in this study.

### Diagnosis of MetS

Criteria from the National Cholesterol Education Program Adult Treatment Panel III (NCEP-ATPIII) were used to define MetS (12). Subjects who were diagnosed with MetS should exhibit at least three of the following components: central obesity (for NHANES subjects, WC ≥ 102 cm for men and ≥ 88 cm for women; for GGMP subjects, WC ≥ 90 cm for men and ≥ 85 cm for women) (13); elevated FPG (≥ 6.1 mmol/L or previously diagnosed type 2 diabetes mellitus); elevated BP (SBP ≥ 130 mmHg or DBP ≥ 85 mmHg, or on BP-lowering medication); elevated TG levels (≥ 1.70 mmol/L; drug treatment for elevated TG); reduced HDL-c levels (<1.03 mmol/L for men and <1.29 mmol/L for women, or taking medication for reduced HDL-c).

### Statistical Analyses

Categorical variables are expressed using absolute numbers and percentages, while continuous variables are expressed as mean and standard deviation (SD), or median and interquartile range (IQR). The chi-squared test, analysis of variance, or the Kruskal-Wallis test were used to compare differences in characteristics among the four races.

A modified sliding-window-based algorithm (SWAN) was used to depict the trajectories of the prevalence of MetS with age for cross-sectional data (14, 15). First, data based on age at a window of 5 years were collected to estimate the prevalence of MetS and its components at this window. The window was subsequently slid forward every year to obtain continuous values of the prevalence at the middle age of each window. Finally, a cubic regression model [the function: *PRE* = *f* (*age*) = *C* + α^*^age + β^*^age^2^ + γ^*^age^3^] was run by harnessing the values of prevalence and the ages to fit a curve that represented a trajectory of MetS prevalence with aging. The growth rate of the prevalence of MetS in aging was estimated by calculating the derivative of the function *PRE* = *f* (*age*).

Principal component analysis (PCA) was used to investigate general racial disparities in the epidemic of MetS among the four races. Five MetS components, and age and sex were included in the PCA, and two packages (“FactoMineR” and “factoextra”) of R software were used to perform the biplot of both variables and the samples.

Odds ratio (OR) and corresponding 95% confidence interval (CI) was used to estimate the association between MetS and its components applying logistic regression stratified according to race and age. Tests for the linear trend of ORs were performed by entering the median value of each category of age as a continuous variable in a linear model. All statistical analyses and plots were performed using R version 3.5.1. Comparisons were performed using the two-tailed test; differences with *p* < 0.05 were considered to be statistically significant.

## Results

### Descriptive Characteristics

The characteristics of MetS and its components as both categorical and continuous variables across the four races are summarized in Table 1. In total, 28,049 adults, with a mean age of 48.9 years, comprising approximately 49.2% males, with an overall MetS prevalence of 33.4%, were included. Strong racial disparities in both MetS and its components were observed (all *p* < 0.001). Chinese subjects exhibited a significantly lower prevalence of MetS (23.0%) than Mexican Americans (37.3%), non-Hispanic whites (36.8%), and non-Hispanic blacks (36.6%) (*p* < 0.001), whereas the prevalence among the latter three races were non-significant. Among the five MetS components, the prevalence of abdominal obesity, elevated FPG, as well as elevated TG levels was lower in Chinese subjects, which is consistent with MetS; however, the prevalence of elevated BP and low HDL-c level was lower among Chinese subjects than in the other three races.

**Table 1:** Characteristics of participants stratified by race.

	**Overall**	Non-Hispanic White^*^	Non-Hispanic Black^*^	Mexican American^*^	Chinese Han^*^
*n*	28,049	10,327	5,674	5,134	6,914
Age (year)	48.9 ± 18.1	49.7 ± 19.2^b^	46.3 ± 18.4^c^	44.6 ± 18.4^d^	52.7 ± 14.7^a^
Sex, male, *n* (%)	13,788 (49.2)	5,187(50.2)^b^	2,978 (52.5)^a^	2,611 (50.9)^b^	3,102 (44.9)^c^
BMI (kg/m^2^)	27.9 ± 6.9	28.8 ± 6.9^c^	30.4 ± 8.1^a^	29.3 ± 6.2^b^	23.4 ± 3.5^d^
Waist circumference (cm)	94.6 ± 17.4	99.7 ± 16.9^a^	99.7 ± 18.2^a^	98.6 ± 14.7^b^	80.3 ± 9.9^c^
FPG (mmol/L)	5.8 ± 1.9	5.8 ± 1.6^c^	5.9 ± 2.3^b^	6.1 ± 2.3^a^	5.6± 1.7^d^
SBP	102.2 ± 24.2	91.3 ± 14.4^c^	94.6 ± 16.0^b^	91.2 ± 14.9^c^	131.7 ± 21.7^a^
DBP	56.1 ± 13.7	51.5 ± 9.5^c^	52.8 ± 10.7^b^	50.9 ± 9.3^d^	77.6 ± 11.5^a^
TG	1.2 (0.8, 1.7)	1.3 (0.9, 1.9)^b^	0.9 (0.7,1.3)^d^	1.4 (0.9, 2.0)^a^	1.1 (0.7, 1.6)^c^
HDL-c	1.3 ± 0.4	1.4 ± 0.4^a^	1.4 ± 0.4^b^	1.3 ± 0.4^c^	1.3 ± 0.5^d^
MetS	9,377 (33.4)	3,801 (36.8)^a^	2,074 (36.6)^a^	1,914 (37.3)^a^	1,588 (23.0)^b^
Abdominal obesity, *n* (%)	13,448 (47.9)	5,770 (55.9)^a^	3,167 (55.8)^a^	2,864 (55.8)^a^	1,647 (23.8)^b^
Elevated FPG, *n* (%)	7,973 (28.4)	2,992 (29.0)^b^	1,930 (34.0)^a^	1,835 (35.7)^a^	1,216 (17.6)^c^
Elevated BP, *n* (%)	10,978 (39.1)	3,642 (35.3)^c^	2,490 (43.9)^b^	1,426 (27.8)^d^	3,420 (49.5)^a^
Elevated TG, *n* (%)	11,129 (39.7)	5,108 (49.5)^a^	2,062 (36.3)^c^	2,391 (46.6)^b^	1,568 (22.7)^d^
Reduced HDL-c, *n* (%)	9,314 (33.2)	3,202 (31.0)	1,343 (23.7)	1,864 (36.3)	2,905 (42.0)

*BMI, body mass index; FPG, fasting plasma glucose; SBP, systolic blood pressure; DBP, diastolic blood pressure; TG, triglycerides; HDL-c, high-density lipid cholesterol; MetS, metabolic syndrome; BP, blood pressure.*

* *P-values for the comparison of all covariates among the four races were < 0.001;^a−d^pairwise comparison among the four races; different letters represent significantly different levels between two races*.

### Prevalence of MetS and Its Growth Rate With Increased Age

**Figure 1:**
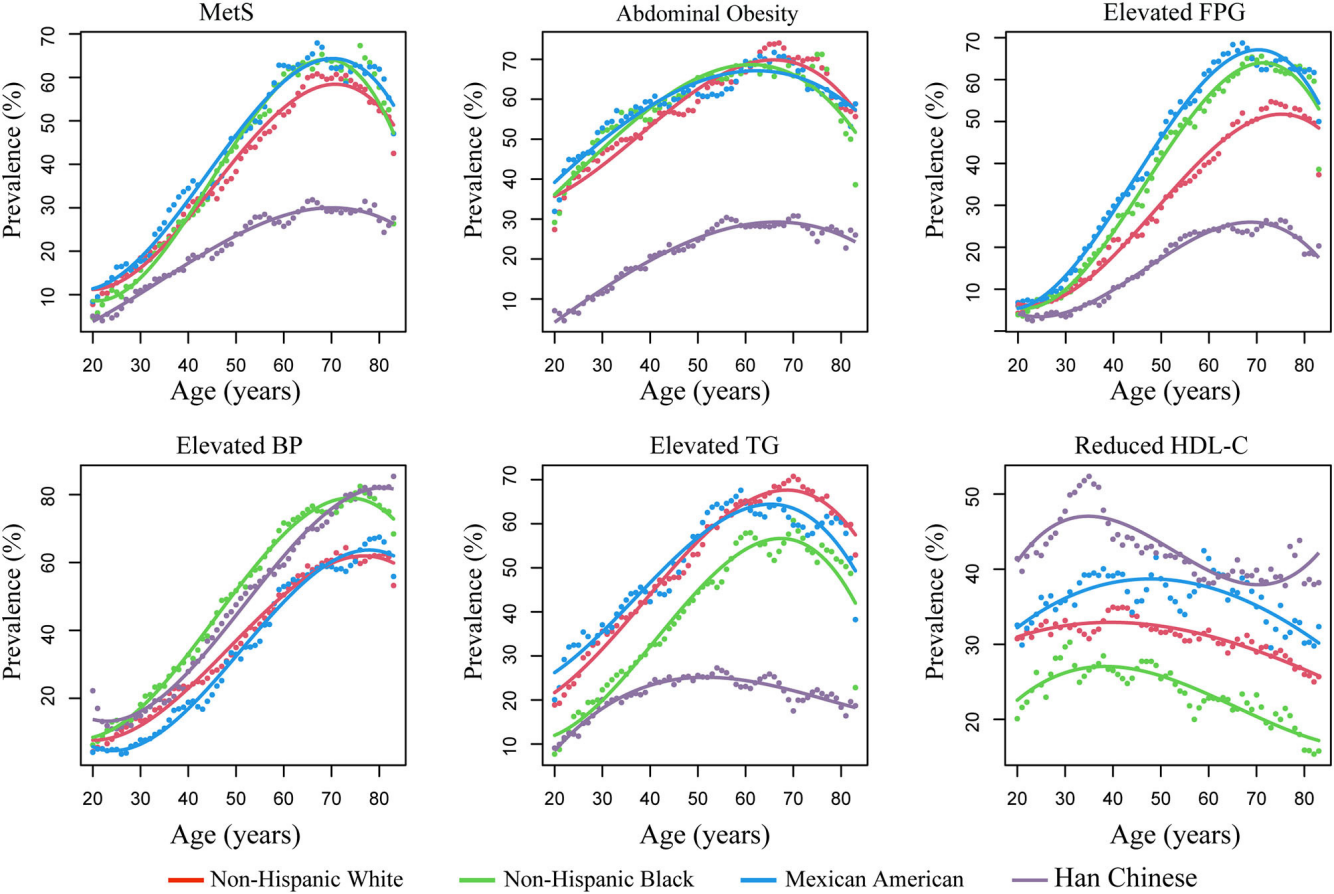
The prevalence of metabolic syndrome (MetS) and its components with age across different races. Prevalence was estimated using a SWAN algorithm (shown as dots). The trajectory of the prevalence of MetS with age was fitted by cubic regressions (shown as lines). FPG, fasting plasma glucose; BP, blood pressure; TG, triglycerides; HDL-c, high-density lipid cholesterol; SWAN, sliding-window-based algorithm.

The trajectories of the prevalence of MetS and its components with increased age across the four races are illustrated in Figure 1. Generally, the trajectories of non-Hispanic whites, non-Hispanic blacks, and Mexican Americans were similar, but were markedly different from that in Chinese subjects. The Chinese subjects demonstrated a lower prevalence of MetS, abdominal obesity, elevated FPG, and elevated TG than non-Hispanic whites, non-Hispanic blacks, and Mexican Americans at all ages, but had a higher prevalence of HDL-c and elevated BP than the other races. This trend persisted after controlling for sex and dividing (i.e., stratifying) subjects into different age categories (Supplementary Figure 1 and Supplementary Table 1).

**Figure 2:**
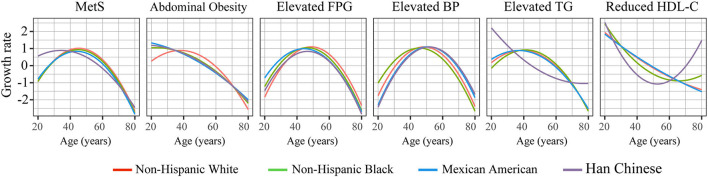
The growth speed of the prevalence of MetS and its components with increased age. The speed at each age was estimated according to the cubic regression.

Significant non-linearity was observed for the prevalence of MetS and its components with increased age; the prevalence changed in “U/J” shapes by applying cubic regressions (*P* for non-linearity <0.05) (Figure 1 and Supplementary Table 2). The prevalence of MetS increased continuously in young and middle-age subjects, flattened, and even decreased in the elderly. Therefore, the growth rate at each age was estimated according to the fitted curves of the cubic regressions (Figure 2). Results revealed that, among Chinese subjects, the growth rate of MetS prevalence continued to increase and reached maximum speed at ~40 years of age, while in the other three races, the maximum growth rate was found at ~50 years. In addition, MetS prevalence was found to start to decrease at ~70 years of age in all races (Figure 1). This trend could be the result of decreased prevalence of four MetS components, which include abdominal obesity (decreased at 60 years), elevated FPG (decreased at 70 years), elevated TG (decreased at 50 years in Chinese subjects and 65 years in the other races), and reduced HDL-c (decreased at 50 years in Mexican Americans and ~40 years in the other races).

### Associations Between MetS and Its Components in Different Age Categories

**Figure 3:**
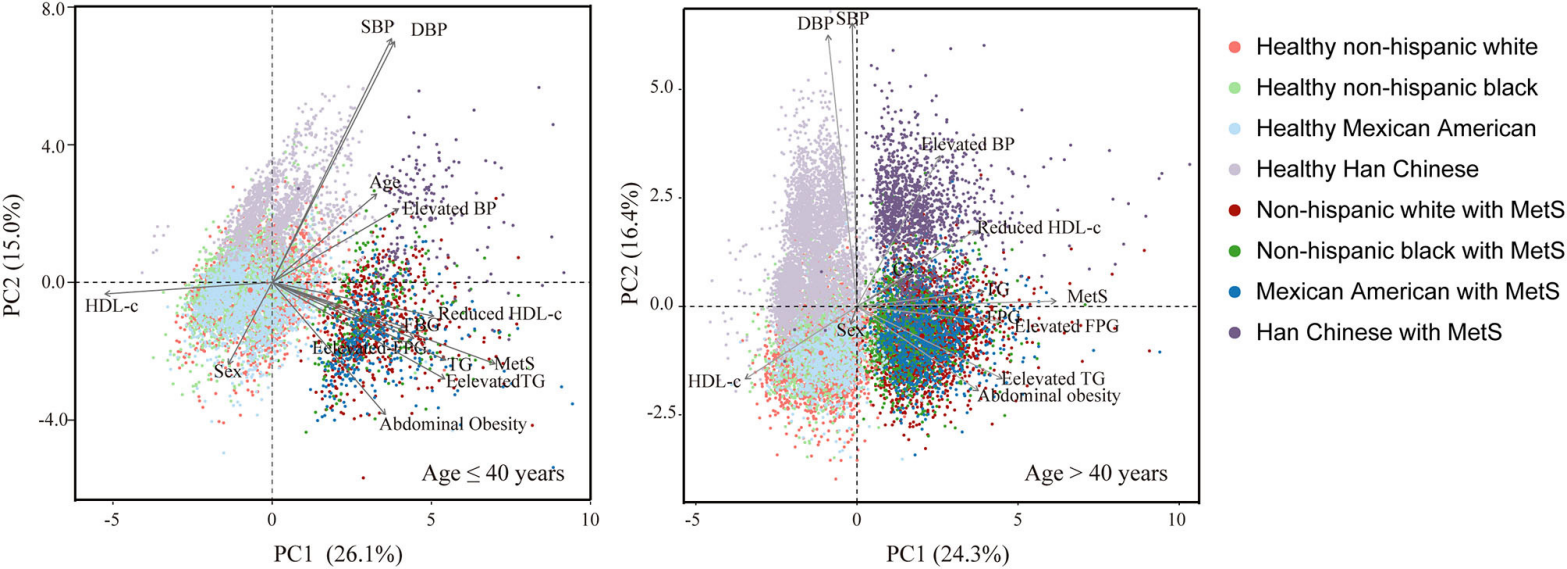
Comparison of components of MetS between non-Hispanic whites, non-Hispanic blacks, Mexican Americans, and Han Chinese according to principal component analysis stratified by age (cut-off age = 40 years).

PCA analysis revealed strong racial disparities in the epidemic of MetS and its components between Chinese and American subjects (Figure 3). Among the five components, abdominal obesity and blood pressure contributed most to the differences between Chinese and the other subjects; however, its contribution to MetS was less than the other components (Supplementary Table 3). The components' contribution to MetS were different between young (age ≤ 40 years) and old (age > 40 years) subjects. In young adults, the most prominent contributors to MetS were elevated TG levels and abdominal obesity, whereas, in older subjects, elevated TG and FPG levels contributed the most to MetS.

To further investigate racial disparities in the associations between MetS and its components with increased age, each component's OR and corresponding 95% CI was estimated for their association with MetS in five age categories (Table 2). For non-Hispanic whites, abdominal obesity was the most important risk factor for MetS (OR 21.34 [95% CI 18.73–24.41]) and persisted in most of the age categories, while for both Mexican American and Chinese subjects, elevated TG level was the most associated component (OR 20.85 [95% CI 17.66–24.71 and 19.84 [17.23–22.89], respectively). The most important MetS component for non-Hispanic black subjects was elevated FPG (OR 25.71 [95% CI 22.13–29.96]); however, it only persisted in subjects 50–70 years of age. Of note, although age affected racial disparities in the associations of MetS with its risk factors, there were consistent results in all subjects <40 years of age, in whom abdominal obesity and elevated TG were the two components most strongly associated with MetS.

**Table 2:** Odds ratios (95% confidence intervals) for the associations between MetS and its components.

	**Overall**	**Age groups**					***P* for trend**
**(l0ptr0pt)3-7**		** <40 years**	**40–49 years**	**50–59 years**	**60–69 years**	**70–85 years**	
**Non-Hispanic white**							
*n*		3,582	1,622	1,526	1,458	2,103	
Abdominal obesity	21.34 (18.73, 24.41)	31.8 (23.14, 44.79)	23.17 (16.33, 33.81)	21.15 (15.29, 29.89)	19.2 (13.87, 27.08)	11.3 (8.95, 14.36)	0.002
Elevated FPG	17.32 (15.55, 19.32)	15.06 (11.75, 19.39)	11.11 (8.49, 14.64)	16.94 (12.89, 22.5)	15.82 (12.05, 20.97)	10.93 (8.87, 13.53)	0.659
Elevated BP	13.87 (12.58, 15.32)	11.51 (9.22, 14.40)	9.41 (7.34, 12.12)	11.2 (8.83, 14.28)	13.23 (10.30, 17.1)	9.14 (7.43, 11.30)	0.786
Elevated TG	17.4 (15.52, 19.54)	17.75 (14.09, 22.56)	16.32 (12.18, 22.17)	12.84 (9.69, 17.23)	11.09 (8.40, 14.79)	12.22 (9.68, 15.53)	0.048
Reduced HDL-c	7.91 (7.20, 8.69)	17.74 (14.07, 22.57)	19.01 (14.66, 24.83)	13.83 (10.56, 18.3)	12.16 (8.78, 17.21)	13.77 (10.33, 18.68)	0.147
**Non-Hispanic black**							
*n*		2,181	925	856	1,068	644	
Abdominal obesity	18.76 (15.73, 22.48)	49.62 (28.32, 95.62)	18.12 (11.53, 29.56)	12.18 (8.09, 18.75)	13.99 (10.02, 19.78)	14.15 (8.94, 23.01)	0.084
Elevated FPG	25.71 (22.13, 29.96)	23.29 (16.58, 33.03)	14.15 (10.04, 20.23)	22.35 (15.58, 32.62)	17.5 (12.68, 24.49)	10.42 (7.15, 15.4)	0.188
Elevated BP	17.59 (15.28, 20.3)	16.17 (12.06, 21.8)	9.07 (6.63, 12.51)	11.52 (8.26, 16.25)	9.70 (7.03, 13.52)	10.32 (6.50, 16.97)	0.18
Elevated TG	22.04 (18.99, 25.64)	36.72 (26.18, 52.29)	13.79 (9.78, 19.73)	14.1 (9.99, 20.18)	10.41 (7.68, 14.25)	14.62 (9.61, 22.7)	0.116
Reduced HDL-c	4.90 (4.29, 5.60)	15.68 (11.57, 21.51)	8.72 (6.27, 12.25)	6.99 (4.73, 10.61)	7.64 (4.99, 12.21)	8.93 (4.88, 18.03)	0.154
**Mexican American**							
*n*		2,199	821	652	935	527	
Abdominal obesity	12.27 (10.43, 14.48)	33.23 (21.81, 52.93)	13.55 (8.73, 21.71)	10.33 (6.83, 15.96)	6.46 (4.61, 9.13)	5.94 (3.9, 9.18)	0.024
Elevated FPG	16.44 (14.22, 19.06)	12.92 (9.66, 17.37)	12.17 (8.55, 17.57)	9.90 (6.83, 14.60)	11.16 (8.05, 15.65)	8.53 (5.68, 13.01)	0.047
Elevated BP	14.72 (12.66, 17.17)	10.41 (7.39, 14.76)	6.09 (4.22, 8.88)	11.59 (7.82, 17.53)	12.33 (8.91, 17.24)	7.86 (5.27, 11.85)	0.982
Elevated TG	20.84 (17.66, 24.71)	21.14 (15.54, 29.28)	20.42 (13.34, 32.14)	11.88 (7.61, 19.16)	14.65 (10.29, 21.14)	18.56 (11.48, 30.81)	0.422
Reduced HDL-c	6.32 (5.57, 7.18)	20.47 (14.9, 28.71)	9.55 (6.88, 13.38)	11.98 (8.02, 18.33)	7.15 (5.06, 10.29)	7.17 (4.44, 12.06)	0.045
**Han Chinese**							
*n*		1,407	1,312	1,789	1,554	852	
Abdominal obesity	15.3 (13.38, 17.52)	30.61 (20.16, 47.22)	15.75 (11.44, 21.86)	12.32 (9.62, 15.85)	12.07 (9.31, 15.74)	11.85 (8.36, 16.98)	0.074
Elevated FPG	10.77 (9.38, 12.39)	26.7 (15.25, 48.21)	10.54 (7.45, 15.02)	8.01 (6.26, 10.3)	8.62 (6.63, 11.26)	11.77 (8.2, 17.09)	0.199
Elevated BP	8.54 (7.39, 9.91)	12.02 (8.23, 17.7)	9.56 (6.92, 13.4)	6.60 (5.09, 8.65)	5.98 (4.39, 8.28)	10.45 (5.37, 23.53)	0.485
Elevated TG	19.84 (17.23, 22.89)	41.85 (26.28, 69.1)	16.32 (11.76, 22.91)	22.21 (16.94, 29.36)	21.5 (16, 29.19)	17.7 (11.77, 27.19)	0.173
Reduced HDL-c	12.39 (10.71, 14.37)	22.86 (12.7, 45.59)	15.09 (10.37, 22.57)	12.59 (9.7, 16.49)	14.76 (11.23, 19.58)	15.46 (10.66, 22.82)	0.236

*FPG, fasting plasma glucose; TG, triglycerides; HDL-c, high-density lipid cholesterol; BP, blood pressure*.

## Discussion

The present cross-sectional study revealed strong racial disparities in the prevalence of MetS, in the growth speed of the prevalence with increased age, and in the associations of MetS with its components among non-Hispanic white, non-Hispanic black, Mexican American, and Chinese subjects. The prevalence of MetS increased non-linearly with age, during which it increased rapidly before 40–50 years of age. The prominent associations between MetS and its components varied among different races and age groups; however, it was consistent that abdominal obesity and elevated TG level had the strongest associations with MetS in all subjects <40 years of age.

The prevalence of MetS in young adults (i.e., 18–40 years) was lower than that in older individuals (40–80 years); however, the growth in prevalence was faster in the former than the latter. Age is a strong risk factor for the incidence of MetS. Age dependency in the prevalence of MS has been observed in many races (16–18). To our knowledge, this is the first study to address how the growth rate of MetS changes with continuously increased age. Our model indicated a potential cut-off point of age (~40 and 50 years for Chinese and American subjects, respectively) for the primary prevention of MetS. According to our models, the high growth speed for young adults could be explained by the high growth rate of abdominal obesity and dyslipidemia. This result is consistent with a previous cohort study involving an Iranian population, in which after an 8-year follow-up, the prevalence of MetS increased by 1.2–3.7% in older (> 40 years) individuals, while it increased by 4.6–5.9% in young adults (<40 years) (19). Another study documented that percentage body fat demonstrated differences before and after 40 years, while it was nonsignificant in the differences among the age groups 40–64, 65–74, and 75–84 years of age in either males or females (20). After 40 years of age, the aging effect on associations between metabolic disease and its factors, such as T2DM vs. obesity, became particularly evident (9). Aging may augment the risk for MetS through complex pathophysiological mechanisms (21); therefore, 40 years of age may represent a key time point before which primary prevention of MetS should be exercised.

Chinese subjects had a lower prevalence of abdominal obesity and a higher prevalence of reduced HDL-c than non-Hispanic whites, non-Hispanic blacks, and Mexican Americans. This trend persisted in most of the age categories. The characteristics of the two components in Chinese subjects are similar to that in some Asian populations such as Korean and Indonesian populations (15, 16), but showed strong heterogeneity from Malaysians and Indians (17, 18). Although it is remarkable that abdominal obesity was generally far more common in Europeans and American subjects than in Asians, Asian populations develop metabolic complications at lower levels of adiposity than western populations (22). Reasons explaining the difference between the two populations remain poorly understood. Racial disparities in the prevalence of hypertension, elevated TG, and hyperglycemia have not been consistent across previous studies and our results. For example, in European studies, hypertension was the main component (23), while we found Chinese subjects had a comparable or even higher prevalence of hypertension compared with other races in subjects >40 years of age. Comparison of the prevalence of MetS components among studies, races, or countries is difficult because the definition of MetS, diet, age, sex, education, and physical activity vary significantly (24).

Preventing the development of the first MetS component has significant public health benefits because the presence of this component is predictive of the development of MetS (25). Therefore, we focused our attention on the question of which component should be first controlled in young and middle-age adults. To answer this question, we investigated the association between MetS and each component from young adults to the elderly, as well as estimated the prevalence of the components in each age category. Our results demonstrated that, although abdominal obesity and reduced HDL-c level (26) were the two most prominent components before 40 years of age, abdominal obesity and elevated TG were the top two components associated with the incidence of MetS. This result indicated a stronger contribution of hypertriglyceridemia to MetS than that of reduced HDL-c in young adults. A previous review documented that hypertriglyceridemia was the major cause of the other lipid abnormalities because it would lead to delayed clearance of TG-rich lipoproteins and formation of small, dense, low-density lipoprotein (27). As a result, abdominal obesity and hypertriglyceridemia would be the key components of MetS that should be the first targets of intervention in early life.

Our study had several limitations, among which we did not control for common factors, such as physical inactivity, urban residency, smoking, and alcohol consumption, because they were measured according to different criteria in the GGMP and NHANES projects. These confounders also contributed to variations in MetS prevalence and, as a result, may have introduced biases. However, a contradictory study reported that variations observed in the associations of risk factors with MetS among ethnic groups did not materially influence the association of ethnicity with MetS (28). Although we used SWAN analysis to depict how the prevalence of MetS changed with increased age in this cross-sectional investigation, it may not represent the actual trajectory of MetS in the aging process. As such, well-conducted cohort studies should be performed in the future. The prevalence of MetS in the GGMP project could not be generalized to all the Chinese subjects. However, the trend in the growth rate of MetS and its most associated components in younger adults may not be affected because we found consistent results across all races.

## Conclusion

Results of the present study revealed significant racial disparities in the prevalence of MetS and its components; the disparity was also observed in the associations of MetS with its components. The disparity varied with increased age; however, a similar pattern was found in younger subjects, in whom abdominal obesity and hypertriglyceridemia were the two components most associated with MetS.

## Data Availability Statement

The original contributions presented in the study are included in the article/Supplementary Material, further inquiries can be directed to the corresponding author/s.

## Ethics Statement

The two survey protocols were approved by the Institutional Review Board of the Centers for Disease Control (Protocol #2005-06, #2011-17, and #2018-01) and Prevention of America and the Ethical Review Committee of the Chinese Centre for Disease Control and Prevention (No. 201519-A). The patients/participants provided their written informed consent to participate in this study.

## Author Contributions

QZ, HA, and JS: conceptualization. CW and XW: methodology and investigation. PZ and YY: validation. RZ, JS, and CW: formal analysis. CW and XW: resources. CW, RZ, and XW: data curation. RZ: writing—original draft preparation. QZ and HA: writing—review and editing. All authors have read and agreed to the published version of the manuscript.

## Funding

Chinese Academy of Medical Sciences Innovation Fund for Medical Sciences (Grant Number 2021-I2M-C&T-A-019).

## Conflict of Interest

The authors declare that the research was conducted in the absence of any commercial or financial relationships that could be construed as a potential conflict of interest.

## Publisher's Note

All claims expressed in this article are solely those of the authors and do not necessarily represent those of their affiliated organizations, or those of the publisher, the editors and the reviewers. Any product that may be evaluated in this article, or claim that may be made by its manufacturer, is not guaranteed or endorsed by the publisher.
