# Supplementary material for: The Racial Disparities in the Epidemic of Metabolic Syndrome With Increased Age: A Study From 28,049 Chinese and American Adults
Source: Front Public Health. 2022 Feb 1;9:797183. doi: 10.3389/fpubh.2021.797183 (PMC8843927; doi:10.3389/fpubh.2021.797183)
Supplement: Supplementary file 1 [file Data_Sheet_1.docx]

**Supplementary Files**

**Supplementary Table 1** Prevalence of MetS and its components stratified by age and races.

|  |  |  | Age groups |  |  |
| --- | --- | --- | --- | --- | --- |
|  | < 40 | 40-49 | 50-59 | 60-69 | 70-85 |
| Non-Hispanic White |  |  |  |  |  |
| Abdominal obesity | 1487(42.3) | 914(57.3) | 993(64.9) | 1044(73.4) | 1332(68) |
| Elevated FPG | 338(9.4) | 375(23.1) | 538(34.4) | 682(46.8) | 1059(50.4) |
| Elevated BP | 449(12.9) | 441(27.8) | 651(42.2) | 819(56.8) | 1282(62.6) |
| Elevated TG | 1083(30.9) | 779(49.4) | 946(63.1) | 970(70) | 1330(67.4) |
| Reduced HDL-c | 1143(32.1) | 547(33.9) | 495(32.0) | 438(30.4) | 579(27.9) |
| MetS | 579(16.2) | 539(33.2) | 705(45.1) | 830(56.9) | 1148(54.6) |
| Non-Hispanic Black |  |  |  |  |  |
| Abdominal obesity | 934(43.7) | 548(61.2) | 566(68.9) | 726(69.9) | 393(68.6) |
| Elevated FPG | 202(9.3) | 283(30.6) | 411(48) | 643(60.2) | 391(60.7) |
| Elevated BP | 327(15.6) | 385(43.0) | 495(59.1) | 787(74.6) | 496(78.2) |
| Elevated TG | 379(18.0) | 337(38.4) | 419(53.4) | 593(61.2) | 334(59.2) |
| Reduced HDL-c | 532(24.7) | 246(26.9) | 200(23.9) | 245(23.6) | 120(19.2) |
| MetS | 277(12.7) | 322(34.8) | 429(50.1) | 670(62.7) | 376(58.4) |
| Mexican American |  |  |  |  |  |
| Abdominal obesity | 981(45.4) | 493(60.7) | 401(62.6) | 655(72.4) | 334(66.7) |
| Elevated FPG | 256(11.6) | 286(34.8) | 348(53.4) | 618(66.1) | 327(62) |
| Elevated BP | 159(7.5) | 173(21.6) | 247(38.8) | 526(57) | 321(61.8) |
| Elevated TG | 686(31.9) | 391(51) | 415(69.3) | 594(70.4) | 305(66.2) |
| Reduced HDL-c | 778(35.5) | 315(38.5) | 238(36.7) | 366(39.9) | 167(31.9) |
| MetS | 364(16.6) | 297(36.2) | 334(51.2) | 602(64.4) | 317(60.2) |
| Han Chinese |  |  |  |  |  |
| Abdominal obesity | 178(12.7) | 289(22) | 503(28.1) | 443(28.5) | 234(27.5) |
| Elevated FPG | 72(5.1) | 178(13.6) | 391(21.9) | 370(23.9) | 205(24.1) |
| Elevated BP | 239(17.0) | 498(38) | 963(53.8) | 1031(66.3) | 689(80.9) |
| Elevated TG | 253(18.0) | 325(24.8) | 449(25.1) | 371(23.9) | 170(20) |
| Reduced HDL-c | 671(47.7) | 564(43) | 729(40.7) | 610(39.3) | 331(38.8) |
| MetS | 156(11.1) | 257(19.6) | 475(26.6) | 453(29.2) | 247(29.0) |

FPG, fasting plasma glucose; BP, blood pressure; TG, triglycerides; HDL-c, high-density lipid cholesterol; MetS, metabolic syndrome; BP, blood pressure.

**Supplementary Table 2** Cubic regression for the prevalence of MetS and its components with increase age across different races.

| **Components** | **Race** | Coefficients | | | |  | P values of the coefficients | | | | R^2^ for  the regression |
| --- | --- | --- | --- | --- | --- | --- | --- | --- | --- | --- | --- |
|  |  | Intercept | Age | Age^2^ | Age^3^ |  | Intercept | Age | Age^2^ | Age^3^ |  |
| MetS | Non-Hispanic white | 0.350692 | -0.0287 | 0.0009 | -6.41E-06 |  | 7.75E-07 | 6.99E-09 | 1.59E-14 | 1.50E-16 | 0.979 |
| MetS | Non-Hispanic black | 0.516063 | -0.0436 | 0.0013 | -9.14E-06 |  | 7.79E-08 | 1.48E-10 | 9.12E-16 | 7.29E-18 | 0.97 |
| MetS | Mexican American | 0.538172 | -0.0442 | 0.0013 | -9.23E-06 |  | 9.14E-13 | 4.17E-16 | 1.26E-22 | 4.16E-25 | 0.985 |
| MetS | Chinese Han | -0.03308 | 0.00051 | 0.0002 | -1.83E-06 |  | 0.366001 | 0.834273 | 0.00038 | 4.30E-07 | 0.979 |
| Central Obesity | Non-Hispanic white | 0.338572 | -0.0072 | 0.0005 | -4.10E-06 |  | 2.64E-05 | 0.151879 | 3.66E-05 | 5.84E-08 | 0.946 |
| Central Obesity | Non-Hispanic black | 0.210518 | 0.00414 | 0.0002 | -2.96E-06 |  | 0.014405 | 0.462256 | 0.04578 | 0.00019 | 0.908 |
| Central Obesity | Mexican American | 0.31306 | 0.0002 | 0.0003 | -2.98E-06 |  | 2.30E-05 | 0.964461 | 0.00499 | 7.45E-06 | 0.923 |
| Central Obesity | Chinese Han | -0.12655 | 0.00792 | 0.00046 | -1.06E-06 |  | 0.001931 | 0.003639 | 0.39376 | 0.00334 | 0.97 |
| Elevated-FBG | Non-Hispanic white | 0.3299 | -0.0267 | 0.0007 | -4.76E-06 |  | 1.65E-10 | 3.16E-13 | 1.73E-17 | 2.69E-18 | 0.98 |
| Elevated-FBG | Non-Hispanic black | 0.402778 | -0.0347 | 0.001 | -6.55E-06 |  | 2.58E-10 | 5.93E-14 | 4.63E-19 | 2.39E-20 | 0.984 |
| Elevated-FBG | Mexican American | 0.479142 | -0.0413 | 0.0011 | -8.07E-06 |  | 1.62E-15 | 3.49E-20 | 1.86E-26 | 1.74E-28 | 0.989 |
| Elevated-FBG | Chinese Han | 0.312057 | -0.0259 | 0.0007 | -5.16E-06 |  | 1.10E-12 | 3.49E-16 | 6.04E-22 | 3.37E-24 | 0.98 |
| Elevated-BP | Non-Hispanic white | 0.29136 | -0.0241 | 0.0007 | -4.96E-06 |  | 3.64E-08 | 1.17E-10 | 3.91E-17 | 1.06E-17 | 0.993 |
| Elevated-BP | Non-Hispanic black | 0.336006 | -0.0304 | 0.001 | -7.29E-06 |  | 2.86E-11 | 5.32E-16 | 6.56E-26 | 4.73E-28 | 0.997 |
| Elevated-BP | Mexican American | 0.498618 | -0.04 | 0.001 | -6.50E-06 |  | 1.33E-11 | 2.48E-14 | 1.79E-18 | 6.71E-18 | 0.991 |
| Elevated-BP | Chinese Han | 0.541909 | -0.0395 | 0.0011 | -7.13E-06 |  | 1.07E-11 | 6.16E-13 | 3.34E-18 | 3.36E-18 | 0.992 |
| Elevated-TG | Non-Hispanic white | 0.240733 | -0.0175 | 0.0007 | -5.81E-06 |  | 0.000466 | 0.000162 | 2.61E-11 | 1.78E-14 | 0.982 |
| Elevated-TG | Non-Hispanic black | 0.318567 | -0.0274 | 0.0009 | -7.07E-06 |  | 0.002389 | 0.000139 | 8.31E-09 | 6.94E-11 | 0.953 |
| Elevated-TG | Mexican American | 0.373914 | -0.027 | 0.001 | -7.62E-06 |  | 5.84E-07 | 1.16E-07 | 5.39E-15 | 8.21E-19 | 0.975 |
| Elevated-TG | Chinese Han | -0.27482 | 0.0245 | -4E-04 | 1.54E-06 |  | 8.39E-10 | 7.22E-14 | 4.82E-09 | 2.37E-05 | 0.911 |
| Elevated HDL-c | Non-Hispanic white | 0.240733 | -0.0175 | 0.0007 | -5.81E-06 |  | 0.000466 | 0.000162 | 2.61E-11 | 1.78E-14 | 0.982 |
| Elevated HDL-c | Non-Hispanic black | 0.318567 | -0.0274 | 0.0009 | -7.07E-06 |  | 0.002389 | 0.000139 | 8.31E-09 | 6.94E-11 | 0.953 |
| Elevated HDL-c | Mexican American | 0.373914 | -0.027 | 0.001 | -7.62E-06 |  | 5.84E-07 | 1.16E-07 | 5.39E-15 | 8.21E-19 | 0.975 |
| Elevated HDL-c | Chinese Han | -0.27482 | 0.0245 | -4E-04 | 1.54E-06 |  | 8.39E-10 | 7.22E-14 | 4.82E-09 | 2.37E-05 | 0.911 |

MetS, metabolic syndrome; FPG, fasting plasma glucose; BP, blood pressure; TG, triglycerides; HDL-c, high-density lipid cholesterol.

**Supplementary Table 3** Contributions of the variables in PCA analysis

| Variables | <40 years | | |  | ≥40 years | | |
| --- | --- | --- | --- | --- | --- | --- | --- |
|  | PC1 | PC2 | PC1+PC2 |  | PC1 | PC2 | PC1+PC2 |
| Sex | 0.70 | 3.66 | 1.78 |  | 0.02 | 0.15 | 0.08 |
| Age | 4.01 | 4.27 | 4.11 |  | 1.12 | 0.32 | 0.79 |
| SBP | 5.23 | 32.43 | 15.17 |  | 0.01 | 39.82 | 16.05 |
| DBP | 5.50 | 31.64 | 15.05 |  | 0.51 | 36.53 | 15.02 |
| FPG | 6.46 | 1.10 | 4.50 |  | 9.94 | 0.09 | 5.97 |
| HDL-c | 10.26 | 0.07 | 6.54 |  | 7.59 | 2.61 | 5.58 |
| TG | 11.07 | 3.29 | 8.23 |  | 9.81 | 0.11 | 5.90 |
| Abdominal obesity | 4.71 | 9.52 | 6.47 |  | 8.96 | 3.53 | 6.77 |
| Elevated FPG | 7.38 | 1.72 | 5.31 |  | 12.45 | 0.11 | 7.48 |
| Elevated BP | 5.86 | 2.97 | 4.80 |  | 4.20 | 11.25 | 7.04 |
| Reduced HDL-c | 9.60 | 0.62 | 6.32 |  | 8.50 | 2.86 | 6.23 |
| Elevated TG | 10.96 | 5.09 | 8.81 |  | 12.82 | 2.60 | 8.70 |

SBP, systolic blood pressure; DBP, diastolic blood pressure; FPG, fasting plasma glucose; TG, triglycerides; HDL-c, high-density lipid cholesterol; MetS, metabolic syndrome; BP, blood pressure.


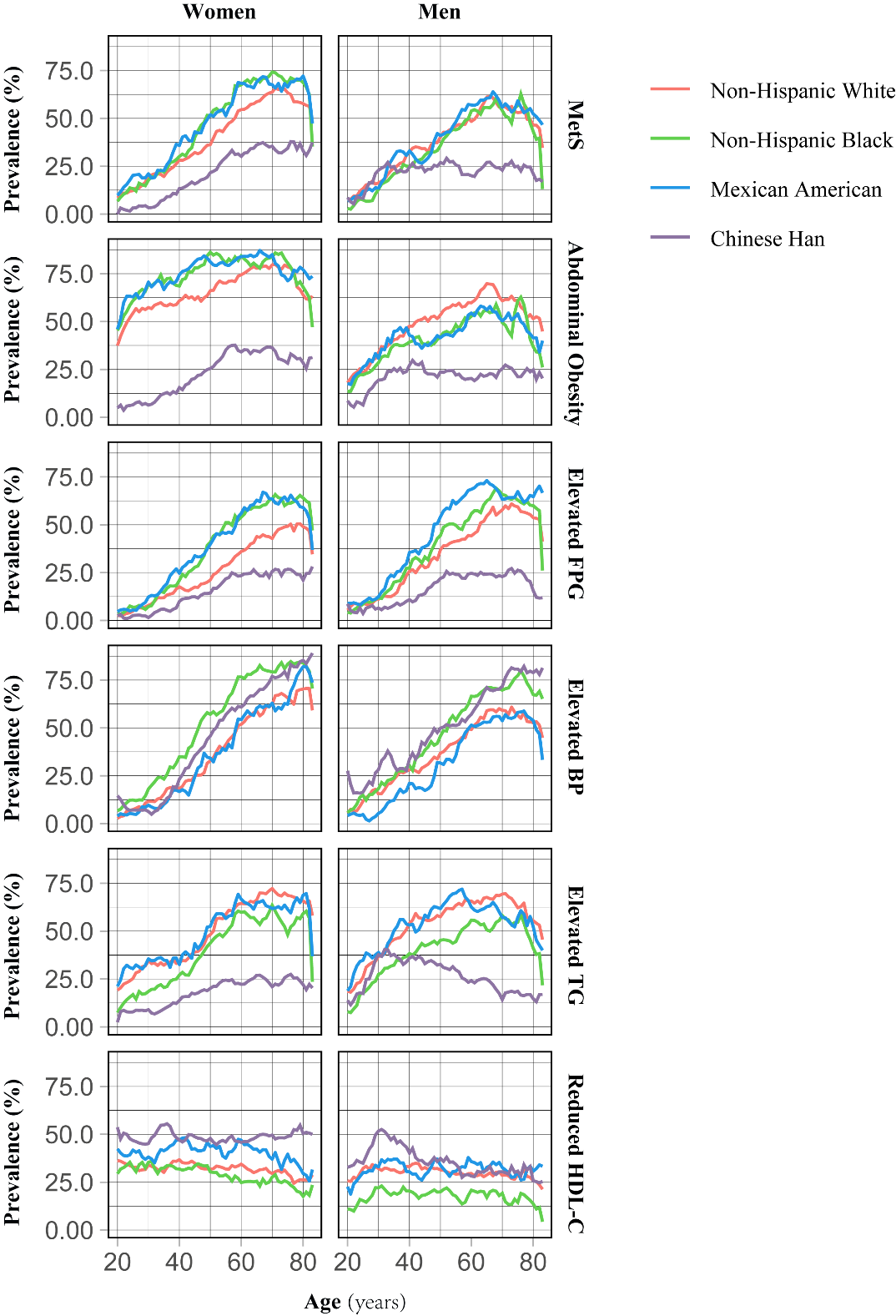


**Supplementary Figure 1** The prevalence of MetS and its components with increased age stratified by sex and races. The prevalence was estimated by SWAN algorithm and showed in the lines of different colors. MetS, metabolic syndrome; FPG, fasting plasma glucose; BP, blood pressure; TG, triglycerides; HDL-c, high-density lipid cholesterol.
